# Oral health in inflammatory bowel disease: the overlooked impact and the potential role of salivary calprotectin

**DOI:** 10.1186/s12903-025-06064-5

**Published:** 2025-05-15

**Authors:** Cláudio Rodrigues, Ana T. P. C. Gomes, Joana Leal, Pedro Pereira, Pedro C. Lopes, Karina Mendes, Maria J. Correia, Nélio Veiga, Nuno Rosa, Caroline Soares, Paula Ministro

**Affiliations:** 1 Gastroenterology Department, Viseu Dão-Lafões Health Unit, Viseu, Portugal; 2 Faculty of Dental Medicine, Center for Interdisciplinary Research in Health, Universidade Católica Portuguesa, Viseu, 3504-505 Portugal; 3 Clinical Pathology Department, Viseu Dão-Lafões Health Unit, Viseu, 3504-509 Portugal; 4 Faculty of Dental Medicine, Universidade Católica Portuguesa, Viseu, 3504-505 Portugal

**Keywords:** Inflammatory bowel disease, Oral health, Salivary calprotectin

## Abstract

**Background:**

Inflammatory Bowel Disease (IBD), a chronic condition characterized by gastrointestinal inflammation, is influenced by genetic and environmental factors. Emerging evidence suggests a “mouth-gut axis,” with the oral cavity reflecting extra-intestinal manifestations of IBD. This study evaluated the oral health status of IBD patients and the potential of salivary calprotectin (SCP) as a biomarker for assessing IBD activity and oral health.

**Methods:**

Oral health was assessed in 100 IBD patients [60 with Crohn’s disease (CD) and 40 with ulcerative colitis (UC)] and 14 controls. Evaluations included the Decayed, Missing, and Filled Teeth (DMFT) Score, Periodontal Diagnosis and the need for dental or prosthetic treatment. Saliva and stool samples were collected to measure SCP and faecal calprotectin (FCP) levels using the Elia Calprotectin 2 Test. IBD activity was evaluated with FCP, the Harvey-Bradshaw Index for CD, and the Partial Mayo Score for UC.

**Results:**

The DMFT index mean was comparable between IBD patients (mean 7.99, SD 7.73) and controls (mean 10.00, SD 6.49). However, periodontal disease was significantly more prevalent in IBD patients (57% in CD, 70% in UC) than in controls (29%), with severe cases (stages III/IV) more frequent in IBD. Additionally, 89% of IBD patients required dental treatment, and 39% needed prosthetic rehabilitation. SCP levels showed no significant correlation with disease activity or oral health status, while FCP correlated with C-reactive protein and erythrocyte sedimentation rate.

**Conclusions:**

This study underscores the need for improved oral health management in IBD patients and suggests that SCP may not be a reliable biomarker for monitoring IBD or periodontal disease.

**Clinical trial number:**

Not applicable.

## Background

Inflammatory bowel disease (IBD) is a chronic, relapsing and remitting disorder of the gastrointestinal tract [1]. IBD primarily includes two distinct inflammatory conditions: Crohn’s disease (CD) and ulcerative colitis (UC). The critical goal in IBD is to restore disease remission as early as possible and to prevent disease progression and bowel damage [2]. While the exact causes of these diseases remain under investigation, current evidence suggests a mix of environmental and genetic factors [3]. The pathophysiology, however, is recognized as an inflammatory response driven by cytokines [3, 4]. This disease is linked to numerous debilitating symptoms, such as urgent diarrhoea, rectal bleeding, vomiting, loss of appetite, and fatigue, which often contribute to poor psychosocial well-being with far-reaching effect [3].

In the last years, there is a growing body of evidence to a potential “mouth-gut axis” in the development of gastrointestinal diseases, including IBD. The oral cavity frequently serves as a site for extra-intestinal manifestations of this disease [5, 6]. This is the primary reason clinicians have started to approach this disease with a broader focus beyond just the intestine.

Despite some contradictory studies that attest that poor oral health seemed to be protective against the development of IBD [7, 8], several other studies [9, 10] provided knowledge on a notable relationship between IBD, both for CD and UC, and increased oral health challenges, such as periodontitis [9], xerostomia [11], and higher incidence of dental caries [12]. Moreover, changes in the oral cavity often appear before intestinal symptoms. IBD can significantly impact oral health, highlighting the need for collaboration between dentists and gastroenterologists to ensure comprehensive patient care and manage the disease [13].

Symptoms have not been established to be the best way of monitoring disease activity since they lack objectivity and correlation with serological and endoscopic parameters [14]. Hence, the goal for treating IBD has shifted in the latest years from symptom control to mucosal healing (MH), which has been demonstrated to be associated with good long-term outcomes. Nevertheless, mucosal healing is best assessed by endoscopy, however this method is invasive, has a high cost and is generally poorly tolerated by patients when performed without sedation [15].

It is in this context that surrogate biomarkers have been sought and proposed to ascertain the inflammatory status and to evaluate disease activity [16, 17]. Calprotectin (CP) is a calcium and zinc binding protein and constitutes about 60% of the neutrophil cytosolic proteins. It is present in a large variety of biologic fluids, such as cerebrospinal fluid, plasma, urine and saliva [16]. Fecal calprotectin (FCP) levels correlate significantly with clinical or endoscopic disease activity in IBD [18]. Its validity as a marker of mucosal inflammation has been extensively studied [16] and hence it has been widely used both in clinical trials and in daily clinical practice, supporting clinical decision-making in patients with suspected or diagnosed IBD [17, 19].

Saliva, a fluid composed of secretions from the major (parotid, submandibular, and sublingual) and minor salivary glands, as well as gingival crevicular fluid, plays key protective and digestive roles [20]. Its collection is non-invasive, easy, and quick collection makes it a convenient alternative to blood sampling [18]. Saliva has shown promise in dentistry and medicine, particularly for diagnosing and monitoring periodontal disease and systemic inflammatory conditions [21]. Numerous biomarkers have been identified in saliva, including cytokines, chemokines, growth factors, and other molecules involved in immune response and inflammation [22].

However, the properties of the saliva of IBD patients are poorly understood [23]. From a conceptual point of view, it is difficult to understand whether calprotectin in saliva can reflect IBD activity, especially since calprotectin may also be increased in patients with periodontal disease [24, 25]. As already mentioned, periodontitis is present in IBD patients, which may threaten the use of saliva calprotectin to assess IBD activity. Nevertheless, the use of saliva as a diagnostic tool is rapidly expanding to include systemic conditions. For IBD, using saliva as a diagnostic fluid would be far more convenient for patients, given its easy collection compared to stool samples. However, data on this topic remains limited. To our knowledge, only four studies have explored this subject, yielding contradictory results. A study involving 23 IBD patients with active disease, 15 resampled after treatment, and 15 controls concluded that salivary calprotectin is elevated in IBD [26]. A recent study by the same author found that the elevation of salivary calprotectin in IBD patients occurs independently of oral disease [27]. Conversely, a study involving 51 patients with active IBD unresponsive to conventional therapy and 51 healthy controls found that salivary calprotectin was significantly lower in both CD and UC patients [23]. Similarly, another study with 63 IBD patients (49 in remission, 7 with active disease) and 11 controls concluded that salivary calprotectin does not correlate with fecal calprotectin or disease activity scores [28]. These conflicting results make it impossible to establish a definitive cut-off value for salivary calprotectin, as it varies depending on testing conditions and methodologies. Studies on SCP levels in IBD patients have used different techniques, such as ELISA, turbidimetric assays, and EliA, leading to inconsistencies in data reporting and challenges in direct comparisons [23, 26, 28]. Some studies quantified SCP without comparing it to faecal calprotectin [26], while others analysed calprotectin across different sample types using varied methods [28]. 

The main goal of this study was to assess the prevalence and severity of oral diseases, specifically periodontitis, dental caries, and the need for oral rehabilitation, in IBD patients and compare with a control group without IBD. Additionally, given the limited and inconsistent data on SCP in IBD patients, this study also aims to explore the role of SCP levels as a biomarker for both disease activity and oral health status in these individuals. The quantification of SCP was performed using the same methodology, equipment, and kits employed for the FCP quantification, with the results being compared to the SCP vs. FCP levels across the samples.

## Methods

### Patient population

A cross-sectional explorative cohort study was performed with consecutive IBD patients visiting the gastroenterology outpatient clinic at Viseu Dão-Lafões Health Unit, Viseu, Portugal. All patients who met the inclusion criteria (age over 18 years old and a diagnosis of IBD according to established criteria) were asked to participate from November 2022 up to December 2023. Recent history of antibiotic treatment (within the preceding month), proton pump inhibitors or non-steroidal anti-inflammatory drugs were exclusion criteria for both patients and controls. In addition, a group of controls without known IBD were recruited among healthcare professionals from our hospital and was chosen based on age-matched healthy individuals without IBD and similar demographic distribution. Controls were aged ≥ 18 years old and were excluded if they had a known diagnosis or complaints of systemic or gastro-intestinal inflammation and if they took the same medications which were exclusion criteria for patients.

### IBD activity

Intestinal disease activity was assessed with FCP levels and the Harvey-Bradshaw Index (HBI) for CD or Partial Mayo score (PMS) for UC. IBD patients were stratified based on a composite criterion for inactive disease using HBI score < 5 for CD [29] or a PMS ≤ 2 for UC [30], and biochemical remission using a FCP cutoff value of 150 mg/Kg, as suggested by recent guidelines [31, 32].

### Oral health assessment

Oral health assessment was achieved by an intraoral clinical examination with the aid of artificial light and oral mirror. Oral examination provided the Decayed, Missing due to caries, and Filled and Teeth Score (DMFT Score) and need for dental treatment and/or prosthetic rehabilitation.

The periodontal status was recorded using a millimeter periodontal probe, that allows a quick and effective assessment of the participant’s periodontal health status. The periodontal diagnosis was based on the criteria established by the 2017 World Workshop on the Classification of Periodontal and Peri-Implant Diseases and Condition [33, 34], allowing to discriminate amongst periodontal health, gingivitis and periodontitis (stage and grade).

### Collection of saliva and stool

In this observational cohort study, unstimulated saliva (around 3mL) was collected through the passive drool technique from adult IBD patients. All patients and controls were submitted to a previous oral examination performed by a dentist. Patients and controls were instructed to refrain from smoking, eating and drinking 30 min prior to collecting saliva. As investigated by Majster et al., fasting does not seem to alter the concentrations of SCP [26], hence this was not a prerequisite. The extracted saliva samples were stored in a freezer at -80 °C for a period of 1 to 4 weeks until analysis. For the correct collection of the stool sample for FCP determination, patients were instructed to collect the first stool sample of the day, avoiding highly liquid or too solid stools. No specific diet restriction was necessary. The stool samples had to be collected within a period of maximum one week, preferentially on the previous day or same day of saliva collection and kept in the refrigerator at 4 °C until the day of processing, generally between 1 and 2 days.

### Calprotectin analysis

Measurement of FCP and SCP concentrations was performed by fluorescence enzyme immunoassay technology (Thermo Fisher Scientific, Uppsala, Sweden), according to the manufacturer’s instructions and the results expressed in milligrams per kilogram.

To determine FCP concentrations, calprotectin (CP) was extracted from fecal samples using EliA™ Stool Extraction Kit, in which each sample incubates with reagent diluent for 10 min. After incubation period, the reagent diluent was analysed using the EliA™ Calprotectin 2 kit and automatically diluted in 1:200 by the equipment. To determine SCP concentrations, serial dilutions using samples from the control group were done until a dilution that fitted the range of the standard curve was achieved. The dilution used was 1:5000. Salivary samples were thawed on ice and diluted 1:50 manually and subsequently samples were analysed using the EliA™ Calprotectin kit, which automatically diluted the samples in 1:100.

### Statistical analysis

Data were presented using means with standard deviation for normally distributed data, median with interquartile range (IQR) for non-normally distributed data and numbers with percentages for categorical data. Normality was tested with a Shapiro-Wilk test. Categorical variables were analyzed using a chi-square test or Fisher exact test. Continuous variables were analyzed using an Independent-Samples T Test for normally distributed variables and a Mann-Whitney U test for non-normally distributed variables. Pearson correlations were used to assess the correlation between normally distributed variables. Spearman’s rank correlation coefficients were used to assess the correlation between non-normally distributed variables. The relationship of SCP and FCP with HBI and PMS scores was determined with a Kendall’s tau-b correlation test. The non-parametric Kruskal–Wallis test was used for multiple comparisons when appropriate, followed by Dunn’s multiple comparisons test. All analyses were performed with IBM SPSS 29.0 (SPSS Inc, Chicago, IL) and *p* values ≤ 0.05 were considered statistically significant.

## Results

Demographics and clinical characteristics of the 100 included IBD patients [60 with Crohn’s Disease (CD) and 40 with Ulcerative Colitis (UC)] and 14 healthy controls are presented in Table 1. There was no difference between the age of controls and IBD patients. The controls had lower prevalence of tobacco use consumption than the IBD patients. 52% of patients were treated with biologic therapy (anti-TNF alfa, vedolizumab and ustekinumab) either monotherapy or combined with immunomodulators (azathioprine and methotrexate). Twelve CD patients had not received any treatment at the time of recruitment, and samples were collected before initiating new therapy. Among them, six were recently diagnosed with CD, while the remaining patients had prior treatments, including immunomodulators and biologic therapy (3 patients), 5-ASA (2 patients), and corticoids (1 patient).

The oral health evaluation showed a higher (although not statistically significant) value of the DMFT index mean in the control group in comparison to IBD patients (mean 7.99, SD 7.73 vs. mean 10.00, SD 6.49). However, the periodontal diagnosis confirms the high incidence of Periodontal Disease (PD) among patients with CD and UC, while a lower occurrence if observed in the control group (Table 1). Most of the patients diagnosed with PD experience the most severe stages of the disease (stages III/IV), with a higher prevalence in IBD groups compared to the control group. Poor oral health status is also evident due to the need for dental treatment, as a significant proportion of IBD patients require dental care, including prosthetic rehabilitation (Table 1). In contrast, the control group shows a lower need for both dental treatment and prosthetic rehabilitation. No ulcers or other oral mucosal manifestations were observed in either IBD patients or the control group during the oral evaluation.

**Table 1:** Demographics, clinical and oral health characteristics of patients

			IBD (*n* = 100)	Controls (*n* = 14)	*p*	CD (*n* = 60)	UC (*n* = 40)	*p*
**Mean age**,** years (SD)**^a^			45 (16)	48 (12)	0.489	42 (17)	49.5 (15)	**0.026**
**Gender**,** F/M**^**b**^			43/ 57	8/6	0.319	22/38	21/19	0.117
**Smoking**^**c**^, **n (%)**	Yes		9 (9)	0 (0)	**< 0.001**	8 (13)	2 (5)	0.081
	No		91 (91)	14 (100)		52 (87)	38 (95)	
**Median disease duration**^d^, **years (IQR)**			8 (10.75)			6 (7.5)	12.5 (10.8)	**0.006**
**Montreal Classification**,** n (%)**	L1 (ileal)					36 (60)		
	L2 (colonic)					6 (10)		
	L3 (ileocolonic)					18 (30)		
	+ L4 (Upper GI)					3 (5)		
	+ p (perianal disease)					15 (25)		
	+ L4 & p					0 (0)		
	B1 (inflammatory)					45 (75)		
	B2 (stenosing)					4 (6.7)		
	B3 (penetrating)					11 (18)		
	E1 (proctitis)						6 (15)	
	E2 (left side)						16 (40)	
	E3 (extensive)						18 (45)	
**Previous Surgery**,** n (%)**	Yes		19 (19)			19 (19)	0 (0)	
	No		81 (81)			81 (81	40 (100)	
**Main previous therapy**,** n (%)**	5-ASA		35 (35)			8 (13.3)	27 (67.5)	
	Immunomodulator		18 (18)			13 (21.7)	5 (12.5)	
	Immunomodulator + biologic		13 (13)			12 (20)	1 (2.5)	
	One biologic		17 (17)			12 (20)	5 (12.5)	
	Two biologics		4 (4)			2 (3.3)	2 (5)	
	≥ Three biologics		2 (2)			2 (3.3)	0 (0)	
	Corticosteroids		2 (2)			2 (3.3)	0 (0)	
	No treatment		9 (9)			9 (15)	0 (0)	
**Current therapy**,** n (%)**	5-ASA		24 (24)			5 (8.3)	19 (48)	
	Immunomodulators		10 (10)			8 (13)	2 (5)	
	Immunomodulators + biologics		11 (11)			8 (13)	3 (7.5)	
	One biologic agent		42 (42)			27 (45)	15 (38)	
	Corticosteroids		1 (1)			0 (0)	1 (2.5)	
	No treatment		12 (12)			12 (20)	0 (0)	
**HBI**,** n (%)**	< 5					50 (83)		
	≥ 5					10 (17)		
**Partial Mayo score**,** n (%)**	≤ 2						34 (85)	
	> 2						6 (15)	
	**Oral Health Status**							
**Mean DMFT**,** (SD)**			7.99 (7.73)	10.00(6.49)		8.41 (8.12)	7.35 (7.17)	
**Periodontal Diagnosis**,** n (%)**	No Periodontal Disease		21 (21)	5 (35.7)		14 (23)	7 (17)	
	Gingivitis		13 (13)	4 (28.5)		10 (17)	3 (7)	
	Edentulous †		4 (4)	0		2 (3.3)	2 (5)	
	Periodontal Disease†† -SI/GB -SII/GB -SIII/GB -SIV/GB		62(62) 10 (10) 19 (19) 24 (24) 9 (9)	4 (29) 0 3 (75) 1 (25) 0		34(57) 7 (11) 7 (11) 14 (23) 6 (10)	28(70) 3 (7) 12 (30) 10 (25) 3 (7)	
**Need for dental treatment**	**Yes** **No**		89(89) 11 (11)	8 (57) 6 (43)		56(93) 4 (7)	33(83) 7 (18)	
**Need for prosthetic rehabilitation (%)**	**Yes** **No**		39 (39) 61 (61)	3 (21) 11 (79)		23 (38) 37 (62)	16 (40) 24 (60)	
	**Biomarkers quantification**							
**Median Fecal Cp**,** mg/Kg**^d^, **(IQR)**			40 (154)	4 (2)	**< 0.001**	37 (162)	45 (142)	0.564
**n (%)**	< 150		74 (74)			44 (73)	30 (75)	
**Median Salivary CP**,** mg/Kg)**^d^, **(IQR)**			216 (422)	298 (232)^*^	0.959	189 (509)	253 (400)	0.844
**Median CRP**,** mg/dL**^**d**^, **(IQR)**			0.1 (0.315)			0.1 (0.32)	0.085 (0.315)	0.862
**Median Ferritin**,** (ng/mL)**^**d**^**(IQR)**			76.5 (84.25)			76 (73)	77 (88.75)	0.647
**Median ESR**,** mm/hr**^**d**^, **(IQR)**			2 (3)			2 (3.75)	3 (3)	0.842

ESR: Erythrocyte sedimentation rate; CD: Crohn’s disease; CP: calprotectin; CRP: C-Reactive Protein; F: Female; HBI: Harvey-Bradshaw Index; IBD: inflammatory bowel disease; M: Male; Mayo PS: Mayo Partial Score; UC: ulcerative colitis; y: years; 5-ASA: 5-aminosalicylic acid

Variables reported as: means with standard deviation for normally distributed data, median with interquartile range (IQR) for non-normally distributed data and numbers with percentages for categorical data; ^a^ Independent-Samples T test; ^b^ Chi2 test, ^c^Fisher exact test ^d^Mann-Whitney U test

CD, Crohn’s Disease; IBD, Inflammatory Bowel Disease; SCP, salivary calprotectin; UC, Ulcerative Colitis

† Edentulous ( no teeth in the oral cavity)

†† Periodontal diagnosis was based on the criteria established by the 2017 World Workshop on the Classification of Periodontal and Peri-Implant Diseases and Condition to discriminate amongst periodontal health, gingivitis and periodontitis (stage and grade). Thus, SI/GB means stage I, Grade B; SII/GB means stage II, Grade B; SIII/GB means stage III, Grade B and SIV/GB means stage IV, Grade B

*Due to the normally distributed data of salivary CP of control group, data are presented as mean (SD)

**Fig. 1:**
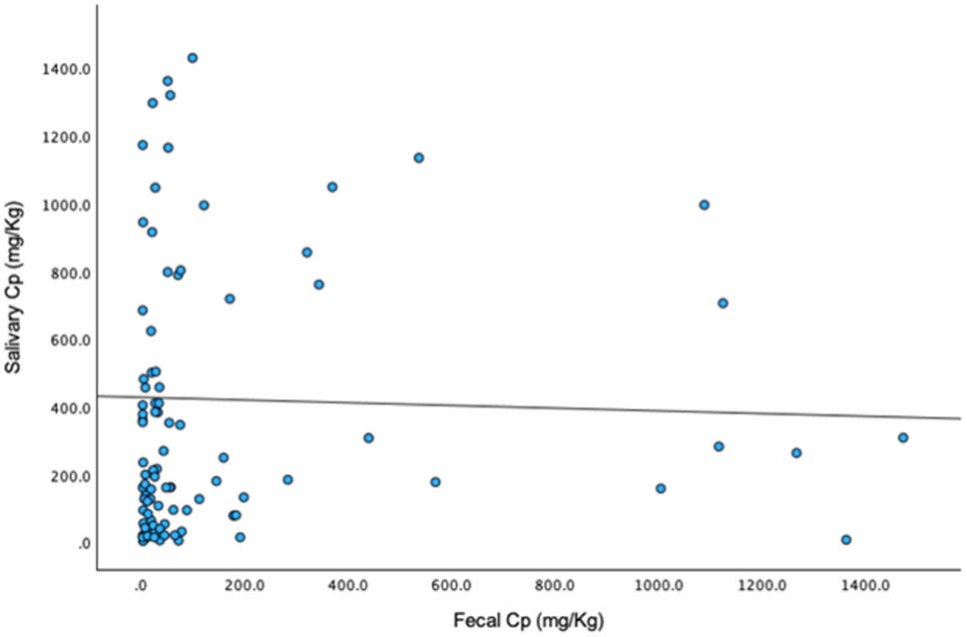
Correlation between salivary and fecal CP in IBD patients. Funnel plot graph

CP levels were assessed in both fecal and saliva samples from all participants and the results are presented in Table 1. In IBD patients, no significant correlation was observed between salivary and fecal CP levels (*p* = 0.358) (Fig. 1). Additionally, no statistical differences in salivary CP levels were found between the control group and IBD patients (*p* = 0.959). When analysing IBD subgroups, salivary and fecal CP levels showed no significant differences between Crohn’s disease and ulcerative colitis (Table 1).

IBD patients were stratified in two groups based on disease activity using a composite criterion (Table 2). A total of 20 patients were classified as having active Crohn’s disease (CD), while 11 were identified with active ulcerative colitis (UC). SCP levels showed no significant differences between patients with active IBD and the control group (*p* = 1.000) or between those with inactive IBD and controls (*p* = 0.942). Furthermore, no statistically significant differences were found in SCP between IBD patients with active disease and in remission (*p* = 0.991), despite the significant difference in FCP, as expected (*p* < 0.001).

When analyzing the impact of treatment, no significant differences were found between treatments groups or no treatment in SCP concentrations (Kruskal–Wallis test, CD (χ^2^ [4] = 7.391, *p* = 0.117; UC group (χ^2^ [4] = 1.780, *p* = 0.7776)). Additionally, no significant correlations (Table 3) were found between SCP and CRP (C-reactive protein) (*p* = 0.685), ferritin (*p* = 0.182) or ESR (erythrocyte sedimentation rate) (*p* = 0.821). Conversely, there was a significant correlation between FCP and CRP (*p* < 0.001) and ESR (*p* = 0.019). Disease activity scores did not correlate significantly with salivary CP (Table 3).

**Table 2:** IBD patients and CD and UC patients stratified into two groups, “remission” and “active disease”

	IBD patients, remission (*n* = 69)			IBD patients, active disease (*n* = 31)		*p*
**Median Salivary Cp**,** mg/Kg (IQR)**	213 (428)			226 (626)		0.991
**Median Fecal Cp**,** mg/Kg (IQR)**	26 (42)			440 (1183)		< 0.001
	**CD** (*n* = 60)			**UC** (*n* = 40)		
	**Remission** **(*n*** **= 40)**	**Active disease** **(*n*** **= 20)**	**p**	**Remission** **(*n*** **= 29)**	**Active disease** **(*n*** **= 11)**	**p**
**Median Salivary Cp**,** mg/Kg (IQR)**	197 (671)	183 (288)	0.50	236 (424)	257 (560)	0.340
**Median Fecal Cp**,** mg/Kg**,** (IQR)**	27 (35)	380 (1158)	< 0.001	20 (49)	1004 (4690)	< 0.001

**Table 3:** Correlations between disease activity scores, CP levels in feces and saliva, CRP, ferritin and ESR in IBD patients

	*N*	*r* statistic	*p* value
**Salivary Cp and Fecal Cp** ^**a**^	100	0.093	0.358
**Salivary Cp and CRP** ^**a**^	100	-0.041	0.685
**Salivary Cp and ferritin** ^**a**^	100	0.023	0.821
**Salivary Cp and ESR** ^**a**^	100	0.023	0.821
**Salivary Cp and HBI** ^**b**^	60	-0.160	0.137
**Salivary Cp and Mayo PS** ^**b**^	40	-0.080	0.544
**Fecal Cp and and CRP** ^**a**^	100	0.516	< 0.001
**Fecal Cp and ferritin** ^**a**^	100	-0.135	0.182
**Fecal Cp and ESR** ^**a**^	100	0.234	0.019
**Fecal Cp and HBI** ^**b**^	60	0.181	0.092
**Fecal Cp and Mayo PS** ^**b**^	40	0.437	0.001

^a^Spearman rho correlation; ^b^Kendall’s tau correlation

In view of the SCP overexpression in IBD patients, the correlation between SCP and the oral health status amongst IBD and control groups was also evaluated (Table 4). The results show no significant differences in the median SCP value between IBD patients (*p* = 0.241) or controls (*p* = 0.192) with or without periodontal disease.

**Table 4:** Correlation between periodontal status and salivary CP

	Periodontal Health	IBD (*n* = 100) Median, (IQR)	*p*	Control (*n* = 14) Mean (SD)	*p*	CD (*n* = 60) Median, (IQR)	*p*	UC (*n* = 40) Median, (IQR)	*p*
**Salivary Cp**,** mg/Kg**	Yes	214 (636)	0.241^a^	394 (236)	0.192^b^	214 (689)	0.294^a^	236 (630)	0.557^a^
	No	217 (436)		226 (219)		171 (456)		257 (435)	

Mann-Whitney U test^a^, Independent-Samples T Test^b^

CD, Crohn’s Disease; IBD, Inflammatory Bowel Disease; SCP, salivary calprotectin; UC, Ulcerative Colitis

## Discussion

### IBD patients suffer from poor oral health conditions

This study has as its primary goal to gain further insights into oral health of IBD patients. The results of the oral health evaluation clearly show that IBD patients have poor oral health, with a high incidence of PD amongst IBD patients, mainly in UC patients. According to *III Estudo Nacional de Prevalência das Doenças Orais– Direção Geral da Saúde* [35], in the ages between 33 and 74, 33% Portuguese population have good periodontal health, which is in line with our assessment of the periodontal disease of the participants in the control group. IBD patients were presented more severe stages of PD, when compared with the control group.

Both PD and IBD are chronic inflammatory conditions driven by a complex interaction between dysbiotic microbiota, dysregulated immune-inflammatory responses in the host and various lifestyle factors. Despite the significant differences in the physical and chemical environments of the mouth and gut, strong correlations have been observed between the microbial compositions of the oral cavity and gut [10, 36]. Most frequent oral diseases reported in IBD patients include mucosal ulceration, mucosal swelling, cobble stoning, orofacial granulomatosis, xerostomia, and an increased risk of dental caries, gingivitis and periodontitis [10, 36, 37].

The relationship between IBD and oral health is supported by a growing body of evidence, which consistently shows that patients with IBD are more likely to experience PD and other oral health problems compared to healthy controls. Studies indicate a higher prevalence of severe periodontitis [7, 38], as observed in our study, mucosal ulcerations, xerostomia, and increased dental caries in individuals with IBD, potentially due to the systemic nature of the disease [39, 40]. The oral microbiome is also suspected to play a key role in IBD pathogenesis, though more research is needed to explore this link, particularly regarding the hygiene hypothesis. Our study highlights the importance of assessing oral health in every IBD patient as a routine procedure due to high prevalence of PD.

### Salivary calprotectin does not correlate with IBD activity nor with periodontal disease

Another goal of this work was to investigate the potential of SCP as a non-invasive biomarker for intestinal disease activity in IBD and/or for oral health status in IBD. Firstly, SCP level was compared with FCP, disease activity scores and inflammatory parameters, namely CRP, ferritin and ESR, in a cohort of 100 patients with IBD. The results showed that there was no correlation between SCP and disease activity measured using FCP and clinical scores of IBD activity. Furthermore, when comparing IBD patients with active and inactive disease and controls, no significant difference in SCP levels was obtained.

Salivary CP has been studied as a potential biomarker in IBD by several authors, however some contradicting results have been found. A study by Nijakowski et al. which included only IBD patients who had already been treated, revealed that SCP levels were significantly lower in CD and UC patients with active disease compared to healthy controls [23]. This is in line with our findings in which patients with IBD also had a lower albeit not significant median SCP level compared to the control group. One hypothesis for such reduction pointed out by the authors is the disrupted host’s oral defense and treatment-induced immunosuppression. However, our results showed no influence of treatment in the values of SCP.

On the contrary, Majster et al. reported a significant increase of SCP in stimulated and unstimulated saliva in patients with active IBD in comparison to healthy controls [26]. Their results showed higher SCP levels in stimulated saliva from newly diagnosed CD patients, however CP levels in unstimulated saliva significantly decreased after treatment in naïve CD patients. The authors highlight this difference, suggesting that SCP may not be a reliable marker for monitoring disease progression, particularly in patients who have already received treatment. However, for the first time, a relation between SCP levels in IBD was attributed to possible subclinical inflammatory responses in the oral cavity as a manifestation of IBD. In fact, 5 years later [27], the same authors evaluated the salivary concentration of CP in IBD patients and related it with the intestinal and oral diseases. This work showed that SCP levels in IBD patients are not influenced by the presence of oral disease, with neutrophils in saliva serving as a source of CP. The authors suggested that oral neutrophils could be impacted by chronic intestinal inflammation, potentially contributing to subclinical oral manifestations of IBD through increased CP secretion. Although salivary CP values in healthy controls tend to be higher than in IBD patients, our results also show no relationship between SCP concentrations and oral health condition, namely PD. Another study focused on the role of SCP in IBD, published in abstract form, supports our findings, showing no correlation between SCP and endoscopic or histologic disease activity scores in 18 patients with CD [41]. Although we did not evaluate endoscopic severity of intestinal disease, we were able to demonstrate that SCP does not correlate with the well-established and reliable biomarker, FCP, nor with disease activity scores.

Several studies demonstrated higher levels of CP in PD patients than in subjects without PD [24]. Although CP has been linked to various inflammatory diseases and disorders, and the increasing evidence supporting its role in the progression of PD, our study found no influence of oral health on SCP levels. Specifically, no correlation was observed between SCP levels and the periodontal status of all the participants. PD is a localized inflammation primarily affecting the gums and supporting structures of the teeth [42], however SCP levels might reflect a broader systemic inflammatory state in chronic inflammatory conditions such as IBD [43, 44]. This data helps to corroborate the weak correlation that exists between SCP, IBD and PD.

Therefore, from our perspective, the potential use of SCP as a biomarker in IBD would require that oral health status does not affect its measurement. Otherwise, assessing oral health before SCP collection would be necessary, which could be impractical in routine clinical practice. This study contributes with new data on the debated role of salivary CP in assessing IBD activity and screening for periodontal disease in IBD patients. To our knowledge, this is the first study in which a team of dentists carried out a comprehensive assessment of the dental health and oral treatment and rehabilitation needs of a large group of IBD patients. Moreover, it was also possible to correlated salivary CP not only with faecal CP and PD, but also with IBD activity indices and biomarkers including ESR, SCP and CRP. Overall, it became clear the importance of accessing oral health in every IBD patients as a routine procedure given the high prevalence of PD.

Nonetheless, this study has some potential limitations. The single-center design may restrict the generalizability of the findings. No formal power calculation was performed prior to the study due to its exploratory nature in assessing the prevalence and severity of oral diseases, and also due to the limited and inconsistent data available on SCP in IBD patients, which impair reliable sample size estimation. Additionally, the number of patients with active disease included in our study was low. While this could be viewed as a drawback, it’s important to remember that IBD is a chronic condition that fluctuates between active phases and periods of remission and the true value of a reliable biomarker lies precisely in its ability to distinguish between these states. Thus, we believe that the small number of patients with active intestinal inflammation did not compromise the validity of our results. While the findings revealed no significant correlation between SCP and disease activity, it is acknowledged that the study may be underpowered to detect subtle associations. As such, this lack of association may not reflect the true absence of effect. Although we did not evaluate endoscopic severity of intestinal disease, we were able to demonstrate that SCP does not correlate with the well-established and reliable biomarker, FCP, nor with disease activity scores. Another potential limitation is the lower number of controls. However, in addition to being matched with the IBD group in terms of age and gender, this group accurately reflects the Portuguese population’s oral health parameters, ensuring that no bias is introduced into the results.

## Conclusion

This study provides important insights into the oral health status of patients with IBD, revealing the high incidence of PD, particularly among patients with UC. These findings underscore the need for increased awareness among healthcare providers regarding the oral health challenges faced by these patients.

Despite the small number of patients with active intestinal inflammation, this study did not identify a correlation between SCP and IBD activity, nor between SCP and PD. However, given the limited number of patients with active disease and the absence of formal power calculation, the possibility that this lack of association reflects insufficient statistical power rather than a true lack of association cannot be excluded. Yet, these findings indicate that SCP may not be a reliable marker for monitoring either condition. While SCP holds promise as a non-invasive biomarker, its clinical utility in IBD remains uncertain due to the influence of other inflammatory factors. Nevertheless, this work contributes to the growing body of literature evaluating non-invasive biomarkers for IBD. Additionally, it underscores the need for further investigation into the potential factors influencing salivary biomarker variability.

Thus, future research should continue exploring the connections between oral and intestinal health in IBD to improve diagnostic and management strategies.

## Data Availability

The data used to generate and support the findings of this study are available from the corresponding author upon request.

## Abbreviations

CD: Crohn’s disease
CP: Calprotectin
CRP: C-reactive protein
DMFT: Decayed, missing, and filled teeth
ESR: Erythrocyte sedimentation rate
FCP: Faecal calprotectin
HBI: Harvey-bradshaw index
IBD: Inflammatory bowel disease
IQR: Interquartile range
MH: Mucosal healing
PD: Periodontal disease
PMS: Partial mayo score
SCP: Salivary calprotectin
UC: Ulcerative colitis
